# Co-exposure of antiretroviral therapy and nicotine induces brain metabolic impairments in a mouse model

**DOI:** 10.1515/nipt-2025-0006

**Published:** 2025-05-22

**Authors:** Gabriel C. Gauthier, Emma G. Foster, Mariano G. Uberti, Balasrinivasa R. Sajja, Aditya N. Bade, Yutong Liu

**Affiliations:** Department of Radiology, 12284 University of Nebraska Medical Center, Omaha, NE, USA; Department of Pharmacology and Experimental Neuroscience, 12284 University of Nebraska Medical Center, Omaha, NE, USA

**Keywords:** antiretroviral therapy, nicotine, chemical exchange saturation transfer, magnetic resonance imaging, magnetic resonance spectroscopy, human immunodeficient virus

## Abstract

**Objectives:**

Anti-retroviral therapy (ART) drastically improves human immunodeficiency virus type 1 (HIV-1) outcomes, but may induce adverse neurochemical changes. Interactive effects of ART with recreational drugs are unknown. Notably, people living with HIV-1 (PLWH) smoke at twice the rate of the general population and are more prone to tobacco-linked illness. Thus, chemical exchange saturation transfer (CEST) magnetic resonance imaging (MRI) was employed to investigate potential ART-nicotine co-morbid neuro-metabolomic influence.

**Methods:**

16 healthy, male, C57BL/6 mice were divided into four groups: vehicle-treatment, ART-treatment, nicotine-treatment, and ART-nicotine co-treatment. CEST-MRI was performed at day 12 following daily treatments to determine effects on neurometabolic profiles. Magnetic resonance spectroscopy (MRS) was used to contextualize metabolic outcomes.

**Results:**

CEST-MRI detected significantly lower 3 ppm contrast in ART, nicotine, and co-treatment groups, suggesting ART- and nicotine-linked glutamate alteration. Co-treatment induced significantly higher hippocampal nuclear Overhauser effects (NOE) compared to ART-treatment, whereas individual treatments lacked effect on NOE, indicating adverse effect on membrane lipids. MRS confirmed CEST findings of membrane turnover, detecting significantly lower hippocampal total choline across all groups compared to controls.

**Conclusions:**

CEST-MRI detects adverse neuro-metabolomic alterations induced by ART- and nicotine-exposure. This warrants investigation with HIV-1-infection to assess potential influences of co-exposure on PLWH cognition.

## Introduction

Consistent use of antiretroviral therapy (ART) is required for the effective treatment of human immunodeficiency virus type-1 (HIV-1) [1], 2]. However, while ART suppresses viral replication, the prevalence of HIV-associated neurocognitive disorders (HAND) in people living with HIV (PLWH) remains high at around 42.6 % [3]. Long-term treatment has been speculated to introduce inherent neurotoxic effects and metabolic dysregulation that contribute to adverse cognitive outcomes, but this is not adequately understood [1], 2]. Similarly, neurochemical alteration of cognition occurs with tobacco consumption, disproportionately impacting PLWH, who smoke at twice the rate of the general population [4]. Tobacco independently alters neurological function and metabolism in complicated ways. Though nicotine exhibits anti-inflammatory properties in microglial cells [5] and plays a role in cholinergic anti-inflammatory pathways [6], it also induces well-demonstrated negative neurological effects, including potential oxidative stress, neurotransmission modulation, and neuroplasticity alteration [7], 8]. As such, there is a critical knowledge gap surrounding fundamental ART-tobacco interactions in the central nervous system (CNS) [9]. Disentangling these interactions is critical to understand whether concurrent exposure of tobacco and ART induce neurotoxicity in PLWH [2], 9].

To date, several MRI methods have been utilized to study different aspects of brain pathology in PLWH. Magnetic resonance spectroscopy (MRS) has identified metabolic hallmarks of HAND, including reduced glutamate (GLU) levels, heightened total choline (tCHO), and reduced N-acetylaspartate (NAA) in PLWH [1]. However, MRS lacks the spatial resolution to map region-wide metabolic changes. Chemical exchange saturation transfer (CEST) magnetic resonance imaging (MRI) addresses this limitation by detecting low-concentration metabolites including GLU, creatine (CR), and macromolecules linked to nuclear Overhauser effects (NOE) with high spatial precision [10]. In this proof-of-concept study, we employed both CEST-MRI and MRS to investigate how ART and nicotine, alone or combined, affect the neuro-metabolic profile using normal C57BL/6 mice. Herein, the focus was to determine potential ART-nicotine co-morbid influences on neuro-health in HIV-1-negative settings. This exclusion of HIV-1 from was intentional, serving as initial confirm of co-morbid influence of ART and nicotine on neuro-metabolome and CEST-MRI’s sensitivity towards detecting these adverse events. This study will set the stage for future investigation of the implications of ART and nicotine co-morbid influence in the setting of HIV-1 infection and whether and how such complex interactions could influence HAND pathobiology. We hypothesize that CEST-MRI with high sensitivity detects ART-nicotine co-exposure induced brain region-specific metabolic alterations.

## Materials and methods

### Animal study approval

This study utilized male C57BL/6 mice, aged 12–14 weeks. All studies were approved by the University of Nebraska Medical Center (UNMC) Institutional Animal Care and Use Committee (IACUC) in accordance with the Guide for the Care and Use of Laboratory Animals.

### Study groups

16 C57BL/6 mice were divided into four groups. Three groups were administered daily with antiretroviral therapy (ART) and/or nicotine for 11 days, while controls received only the corresponding drug delivery vehicles. Group 1 mice (n=4) were administered ART by oral gavage. The ART regimen was comprised of a combination of tenofovir disoproxil fumarate (TDF) at 250 mg/kg (mouse-weight), lamivudine (3TC) at 250 mg/kg (mouse-weight), and dolutegravir (DTG) at 50 mg/kg (mouse-weight). These dosages correspond to five times the human equivalent dose (HED) of ART regimen. ART suspension was prepared in a vehicle of 0.2 % hydroxymethyl cellulose and 0.1 % Tween 80 in sterile water. Group 2 mice (n=4) were administered nicotine through intraperitoneal (i.p.) injection at 2 mg/kg (mouse-weight). Nicotine solution was prepared in sterile phosphate-buffered saline (PBS). Group 3 mice (n=4) received both ART and nicotine, as described above. Group 4 mice (n=4) were administered ART-vehicle and PBS.

### Magnetic resonance imaging

24 h after final treatment, mice underwent CEST-MRI and MRS. CEST data were acquired using a RARE sequence with saturation frequency range of −5 to +5 ppm, with offset step=0.2 ppm. Acquisition parameters included: single slice thickness=0.5 mm, saturation RF amplitude=2 µT, and saturation duration=1 s. Main magnetic field (B0) inhomogeneity was corrected using water saturation shift referencing (WASSR). CEST data were analyzed using 5-pool Lorentzian fitting, quantifying contrasts at 2 ppm (CR), 3 ppm (GLU) and −3.5 ppm (NOE). Water and magnetization transfer contrast were also included. CEST values were derived from area-under-curve (AUC) calculations of Lorentzian functions in five regional slices: cortex, hippocampus, piriform cortex, thalamus, and whole brain. These regions were selected for their roles in coordinating higher-level cognition, regulation of memory, sensory integration, and olfaction. Single-voxel MRS was carried out via semiLASER sequence (TR/TE=4,000/40 ms) in the cortex (5×0.75×1.5 mm^3^) and hippocampus (6×1×1.5 mm^3^) with variable power RF pulses with optimized relaxation delays (VAPOR) for water suppression. Data were quantified using LCModel, with metabolite concentrations normalized to in-voxel water content.

### Statistical analysis

Comparisons between groups were conducted with ordinary one-way ANOVA and two-tailed t-tests using GraphPad’s Prism (La Jolla, CA). Outlier values were defined outside 150 % interquartile range, excluding a single Nicotine-treated mouse. Data were expressed as mean±standard error of the mean (SEM) with four biological replicates. Statistical significance (p<0.05) is denoted by an asterisk (*), while a statistical trend (0.05<p<0.1) is denoted by a hash symbol (#).

## Results

### CEST-MRI detects ART-and-nicotine-linked glutamate impairments

**Figure 1:**
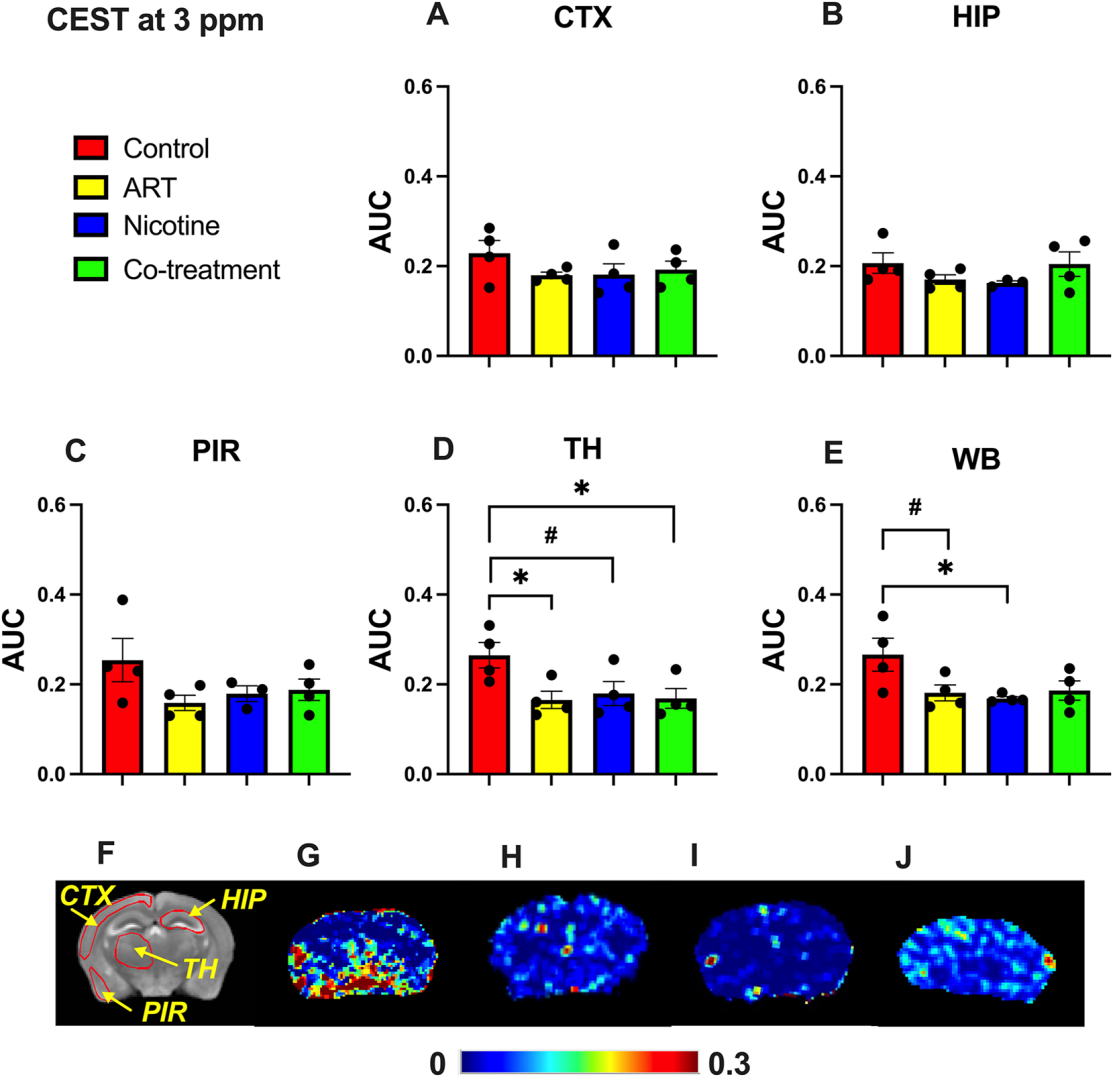
Regional 3 ppm CEST contrast. The average CEST signal in the (A) CTX, (B) HIP, (C) PIR, (D) TH, and (E) WB regions. Statistical significance was denoted as follows: #: 0.05<p<0.1, *: 0.01<p<0.05. The anatomical map (F) provides ROI reference for the provided heatmaps (G–J), which depict the contrast in the control, ART-treatment, nicotine-treatment, and Co-treatment groups, respectively.

Regional 3 ppm CEST (glutamate) contrasts were quantified in slices of the cortex, hippocampus, piriform cortex, thalamus, and whole brain (Figure 1A–E). Each plot represents the AUC of the 3 ppm Lorentzian function. Significantly lower 3 ppm contrast was observed in the thalamus (p=0.027) of ART-treated mice compared to controls, with a trend of lower contrast in the whole brain (p=0.089). In nicotine-treated mice, significantly lower contrast was observed in the whole brain (p=0.036) compared to controls, with a trend of lower contrast in the thalamus (p=0.070). In co-treatment mice, significantly lower contrast was observed in the thalamus (p=0.036) compared to controls. 3 ppm CEST contrast heatmaps of representative mouse brains are provided (Figure 1F–J), illustrating treatment glutamatergic impact. Significantly higher contrast is observed in the thalamus of control mice compared to all other groups. These results indicate that both ART and nicotine contribution to thalamic GLU reduction without synergistic glutamatergic effects.

### CEST-MRI detects co-treatment-linked macromolecular turnover

**Figure 2:**
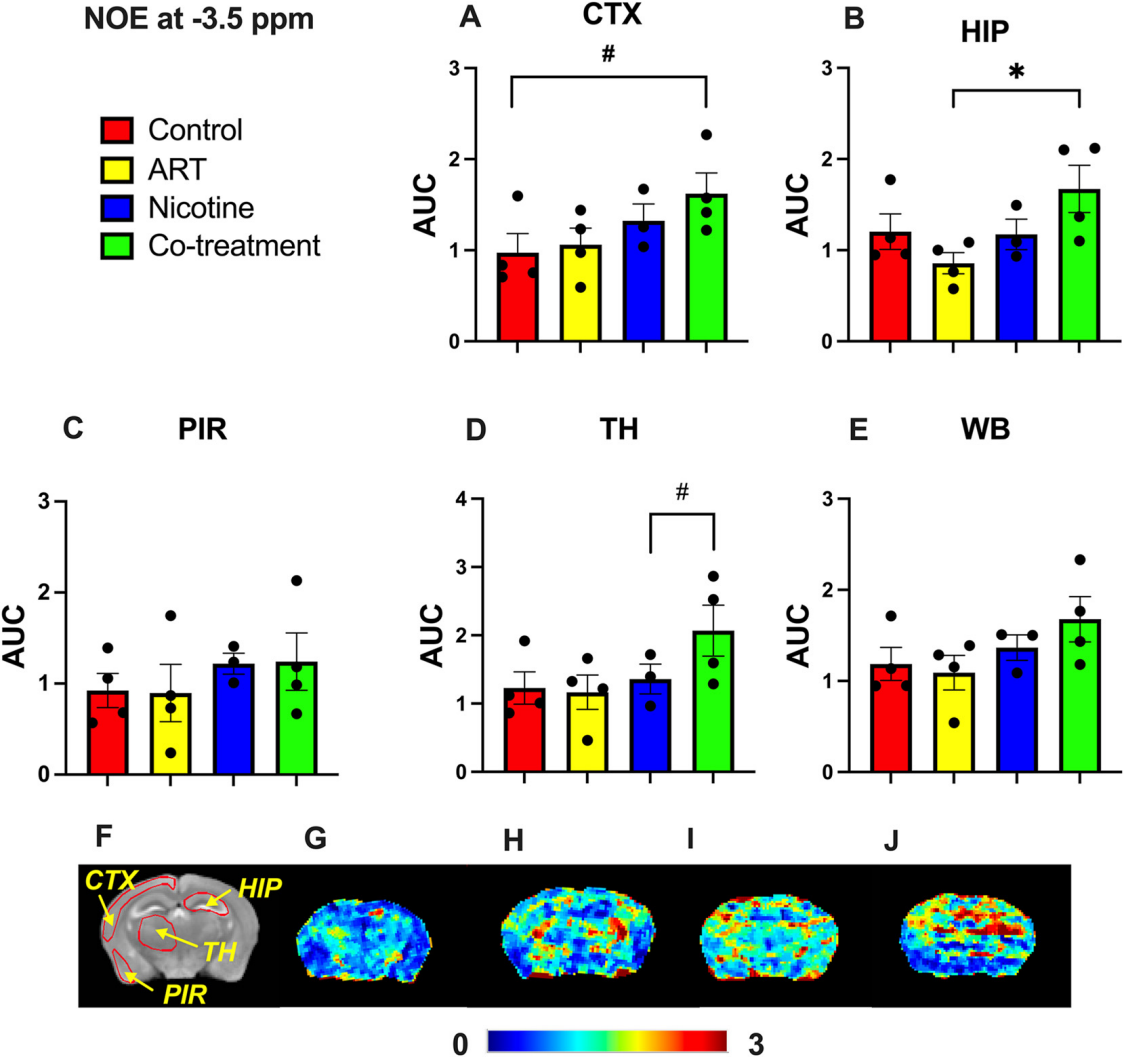
Regional NOE contrast. The average NOE signal in the (A) CTX, (B) HIP, (C) PIR, (D) TH, and (E) WB regions. Statistical significance was denoted as follows: #: 0.05<p<0.1, *: 0.01<p<0.05. The anatomical map (F) provides ROI reference for the provided heatmaps (G–J), which depict the CEST contrast in the control, ART, nicotine, and Co-treatment groups, respectively.

Regional NOE at −3.5 ppm, associated with macromolecule presence [11], was quantified (Figure 2A–E). Co-treatment mice showed significantly higher hippocampal NOE (p=0.028) compared to ART-treated mice. Furthermore, trends of higher contrast were observed in the cortex compared to controls (p=0.082) and in the thalamus (p=0.093) compared to nicotine-treated mice. Heatmaps illustrate treatment NOE effects (Figure 2F–J). Higher NOE is observed in the hippocampus and thalamus of co-treatment mice compared to others. These results indicate potential nicotine alteration of ART’s regional macromolecular impact.

### MRS contextualizes CEST-MRI

**Figure 3:**
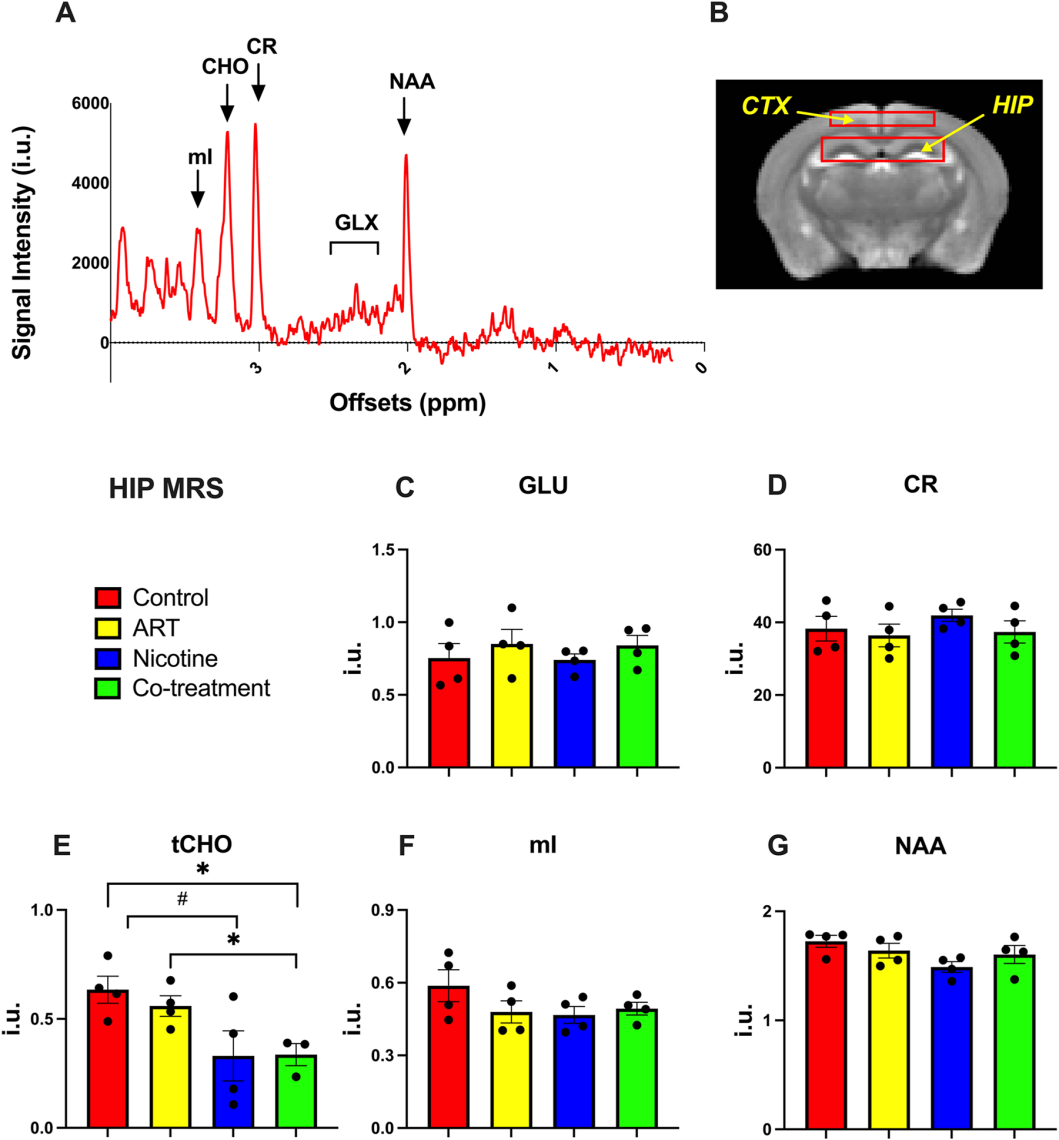
HIP MRS. (A) The representative MRS spectrum of a control mouse. (B) The locations of MRS voxels on CTX and HIP. (C–G) bar graphs comparing the MRS values of GLU, CR, tCHO, mI, and NAA in the HIP. GLU, tCHO, mI, and NAA are reported as ratios to creatine. Statistical significance was denoted as follows: #: 0.05<p<0.1, *: 0.01<p<0.05.

Metabolic concentrations in the cortex and hippocampus were quantified with single-voxel MRS (Figure 3). A control mouse MRS spectrum is shown alongside cortex and hippocampus voxel placements (Figure 3A and B). Significantly lower hippocampal tCHO, associated with membrane turnover [12], was observed in co-treatment mice compared to vehicle-treated (p=0.017) and ART-treated (p=0.024) mice. Additionally, nicotine-treated mice showed trends of lower hippocampal tCHO compared to controls (p=0.059). No significant group-wise differences were observed for cortical tCHO.

## Discussion

This study provides evidence that ART-nicotine interaction induces alterations in GLU (an excitatory neurotransmitter) [13], NOE (linked to lipid membrane turnover and neuroinflammatory processes) [11], and tCHO (a marker of membrane integrity) [12], 14]. This was affirmed using novel application of CEST-MRI. In C57BL/6 mice, CEST-MRI detected significantly lower thalamic GLU (3 ppm) following ART-, nicotine-, and co-treatment. In addition, significantly higher hippocampal NOE (−3.5 ppm) was noted for co-treatment group compared to ART-treatment alone. Further, this observation was contextualized by MRS findings, in which lowered hippocampal tCHO was observed in co-treatment mice.

Generally, deficits in GLU have been attributed to variety of factors, including altered glutamate-glutamine recycling, increased glutamatergic demand, neuronal damage, and mitochondrial inhibition from oxidative-stress [13]. The observed differences in NOE may suggest altered macromolecular composition, consistent with neuroinflammatory processes or membrane injury [15]. While tCHO reduction may indicate diminished inflammation or decreased neurogenesis, the concurrent NOE rise may imply compensatory non-choline macromolecular upregulation (e.g. lipids or proteins) during neuroinflammatory remodeling [11].

Though the observed trends in our data resemble GLU reductions observed in HAND patients [1], 13], further investigation is needed in the settings of HIV-1 infection to clarify the connection between the observed neurochemical changes and cognitive deficits seen in PLWH. Similarly, changes in hippocampal NOE and tCHO potentially indicate neuroinflammation or membrane injury, though biological validations are required to confirm this hypothesis.

A few limitations are recognized. First, our study studies ART exposure in HIV-1-negative rodents, shedding light specifically on ART-nicotine interaction independent of HIV-infection. Despite clarifying ART-nicotine interactions for future studies, HIV-1 exclusion limits immediate translation to PLWH, where HIV-1 pathology synergizes with ART and addiction. Second, a nicotine solution was administered intraperitoneally (bypassing respiratory exposure and the effect of chemical additives), decreasing lung injury and oxidative stress associated with smoking/vaping tobacco, which independently influence neuroinflammation. Third, our ART regimen represents just one treatment plan at supratherapeutic doses; neurotoxicity and drug interactions may differ with other drugs formulations and dosages. Fourth, small group sizes (n=4) and short-term exposure (11 days) may primarily reflect initial treatment response, rather than chronic/cumulative effects relevant to neuropathology in PLWH. Fifth, young male mice do not capture sex-specific neuroimmune responses and aging-related vulnerabilities prevalent in PLWH. Finally, neurochemical changes were not paired with behavioral testing, leaving functional implications of the data unclear. In the future, we will conduct studies incorporating HIV-1-infection, inhalation-based nicotine delivery, and diverse ART regimens to enhance clinical relevance. Longitudinal studies with larger, mixed-sex cohorts and behavioral assessments will be conducted to clarify the progression of neurochemical changes and functional deficits.

## Conclusions

Our findings underscore the utility of CEST-MRI in detecting region-specific neurochemical alterations induced by ART and nicotine. These findings further warrant comprehensive investigation of potential influences of ART-tobacco co-exposure on CNS in animal model with HIV-1-infection to reflect clinical manifestation of neuropathology as it is seen in PLWH.

## Supplementary Material

Supplementary Material Details
