# Supplementary figures and images for: Co-exposure of antiretroviral therapy and nicotine induces brain metabolic impairments in a mouse model
Source: NeuroImmune Pharm Ther. 2025 May 22;4(2):265–71. doi: 10.1515/nipt-2025-0006 (PMC12455577; doi:10.1515/nipt-2025-0006)

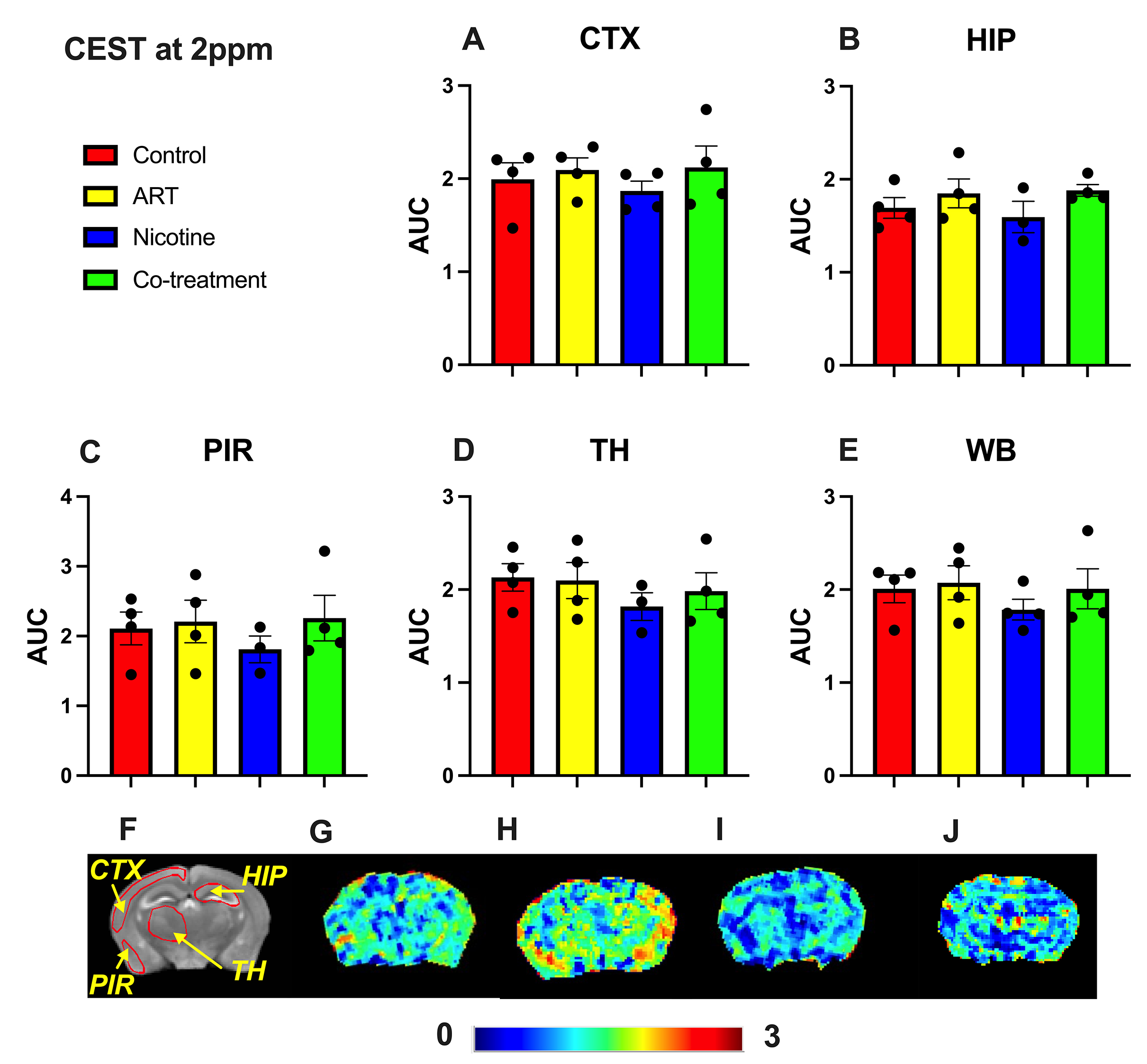

Supplement: Supplementary file 1 — Supplementary Material Details [file j_nipt-2025-0006_suppl_001.jpg]
